# Liposomal Lactoferrin Exerts Antiviral Activity against HCoV-229E and SARS-CoV-2 Pseudoviruses In Vitro

**DOI:** 10.3390/v15040972

**Published:** 2023-04-15

**Authors:** Sabina Andreu, Inés Ripa, Raquel Bello-Morales, José Antonio López-Guerrero

**Affiliations:** 1 Department of Molecular Biology, Universidad Autónoma de Madrid, C/Darwin, 2 Cantoblanco, 28049 Madrid, Spain; ines.ripa@cbm.csic.es (I.R.); raquel.bello-morales@uam.es (R.B.-M.); ja.lopez@uam.es (J.A.L.-G.); 2 Centro de Biología Molecular Severo Ochoa, Spanish National Research Council—Universidad Autónoma de Madrid (CSIC-UAM), C/Nicolás Cabrera, 1 Cantoblanco, 28049 Madrid, Spain

**Keywords:** lactoferrin, liposomal lactoferrin, antivirals, SARS-CoV-2, COVID-19

## Abstract

A limited number of effective therapies are currently available to treat human coronavirus SARS-CoV-2 and other human coronaviruses, which are responsible for nearly a third of global cases of the common cold. The possibility of new emerging coronaviruses demands powerful new antiviral strategies. Lactoferrin is a well-known protein that possesses anti-inflammatory and immunomodulatory activities, and it has previously shown antiviral activity against several viruses, including SARS-CoV-2. To increase this antiviral activity, here we present bovine liposomal lactoferrin. Liposomal encapsulation of the compound was proven to increase permeability, bioavailability, and time release. In the present work, we compare the antiviral activity of free and liposomal bovine lactoferrin against HCoV229E and SARS-CoV-2 in vitro and in human primary bronchial epithelial cells, and we demonstrated that the liposomal form exerts a more potent antiviral activity than its free form at non-cytotoxic doses.

## 1. Introduction

COVID-19 is a disease caused by the severe acute respiratory syndrome coronavirus 2 (SARS-CoV-2) that has led about seven million deaths worldwide [1]. Two other coronaviruses have also posed a serious threat to global health in the last decades: SARS-CoV-1 and MERS-CoV. There are also four common human coronaviruses (HCoV-229E, HcoV-NL63, HcoV-OC43, and HcoV-HKU1 [2]), which are responsible for 15–30% of the global cases of common cold [3]. Despite the advances in vaccination, the use of antivirals for pharmacological treatments against SARS-CoV-2 and other coronaviruses is required [4]. Unfortunately, a limited number of effective therapies against COVID-19 are available, and the possibility of new outbreaks of circulating or emerging coronaviruses makes the search for new antiviral agents against these viruses strongly necessary.

To overcome the need to develop treatments in a very short time, an interesting approach is the reuse of already approved drugs. Based on this approach, we herein present a new format of lactoferrin (Lf), an iron-binding protein found in many mucosal secretions and in neutrophil granules that exhibits multifunctional activities; it is anti-inflammatory [5], anti-oxidative [6], immunomodulatory [7], and presents broad-spectrum antibacterial, antifungal, and antiviral activity [8,9,10]. Regarding antiviral activity, viruses such as herpes simplex virus type 1 (HSV-1), human immunodeficiency virus (HIV), human hepatitis C virus (HCV), human papilloma virus (HPV), respiratory syncytial virus (RSV), Sindbis virus, and Semliki Forest virus, among others, have also been proven to be highly sensitive to human and/or bovine Lf in vitro [8,9,11,12]. In addition, previous studies have investigated the potential in vitro antiviral activity of bovine Lf (bLf) against SARS-CoV-1 [13] and SARS-CoV-2 [14]. In addition, some clinical trials have reported the efficacy of bLf against SARS-CoV-2 [15,16].

bLf is accepted by the Food and Drug Administration (FDA) as ‘generally recognized as safe’ (GRAS), and it is used as a nutritional additive [9]. To improve bioavailability, formulations of bLf based on encapsulation and liposomalization have been tested [15,17,18,19]. Liposomes are spherical vesicles (of a size ranging from 30 nm to 1 µm) which enclose a central aqueous compartment surrounded by one or more phospholipid membranes. They are commonly used as drug carriers of small molecules, as liposomalization enhances many properties, such as their stability, permeability, bioavailability, selectivity, and time release, while reducing systemic toxicity [18,19,20]. In fact, liposomalization of bLf has already been demonstrated to potentiate its anti-inflammatory properties [17].

Here we report the potential antiviral activity of liposomal bovine lactoferrin in vitro against human coronavirus HCoV-229E and SARS-CoV-2 pseudoviruses in comparison to non-liposomal bovine lactoferrin.

## 2. Materials and Methods

### 2.1. Cell Lines

The Huh-7 cell line [21] was generously provided by Dr. Sonia Zúñiga (CNB-CSIC, Madrid, Spain). A549 lung carcinoma cells and human embryonic kidney HEK293T cells native or expressing human ACE2 were generated by lentiviral transduction with vector CSIB and selection in blasticidin S [22]. All cell lines were routinely tested for the absence of mycoplasma.

Cell lines were cultured in low-glucose Dulbecco’s modified Eagle medium (DMEM) (Life Technologies) supplemented with 5% fetal bovine serum (FBS), penicillin (50 U/mL), and streptomycin (50 µg/mL) at 37 °C in a humidified atmosphere of 5% CO_2_.

### 2.2. Lung Tissue

Lung tissues were obtained from patients with no history of COVID-19 and with a recent negative PCR test for SARS-CoV-2 infection undergoing thoracic surgical resection at the Thoracic Surgery Service of the Vall d’Hebron University Hospital (Barcelona, Spain). Cell extraction was performed as described in Grau-Expósito et al., 2022 [23]. Briefly, non-neoplastic tissue areas were dissected into small blocks and digested with collagenase IV (Gibco) and DNase I (Roche) for 30 min at 37 °C and 400 rpm, and they were mechanically digested with a pestle. The resulting cellular suspension was subjected to several filtrations and washes with PBS and was finally resuspended with RPMI 1640 supplemented with 5% FBS, 100 U/mL of penicillin, and 100 ug/mL of streptomycin. Cell number and viability were evaluated with the LUNA automated cell counter (Logos Biosystems, South Korea.

Regarding human lung tissue cells, the study protocol was approved by the Clinical Research Committee (Institutional Review Board number PR(AG)212/2020) from the Vall d’Hebron University Hospital in Barcelona, Spain. Samples were obtained from adults, all of whom provided their written informed consent.

### 2.3. Viruses

HCoV-229E expressing a GFP reporter protein was generously provided by Dr. Volker Thiel from the University of Bern. This virus was propagated in Huh-7 cells for 5 days at 33 °C with 5% CO_2_. The infectious titer of the virus stocks was determined according to the Reed and Muench formula [24] on Huh-7 cell monolayers by the endpoint dilution assay described in Andreu et al., 2021 [25].

Lentiviral particles expressing either the SARS-CoV-2 Spike (St) protein (Wuhan, truncated) or vesicular stomatitis virus (VSV) protein and GFP reporter protein were generated as in Horndler et al., 2021 [22]. Briefly, pseudoviruses were obtained by co-transfecting plasmids pCMVA (gag/pol), p-HR-SIN-GFP, and either a truncated S envelope (pCR3.1-St) or VSV envelope (pMD2.G) using the JetPEI transfection reagent (Polyplus Transfection). Viral supernatants were obtained after 24 and 48 h of transfection and pooled. Polybrene (4 μg/mL) was added to the viral supernatants before the addition to ACE2+HEK293T cells. Cells were centrifuged for 70 min at 2100 rpm at 32 °C and left in culture for 48 h. Finally, cells were resuspended with 5 mM EDTA and fixed for flow cytometry analysis. Both LV-St and LV-VSV were titrated on ACE2+HEK293T cells by analysis of GFP+ cells on a FACSCanto™ II Flow Cytometer (Becton-Dickinson, Franklin Lakes, NJ, USA), and data were processed with FlowJo software (BD, version 10.6.2).

### 2.4. Reagents

Free bLf (FL) or liposomal bLf (LL) encapsulated in a phosphatidyl choline liposome were provided by the Sesderma company (Sesderma S.L, Valencia, Spain). Dilutions were freshly prepared each time.

Lf was encapsulated in positively charged PC-liposomes at the mentioned concentrations. The liposome preparation presented a unimodal size distribution with a diameter between 80 and 150 nm, a polydispersity index below 0.20, and a zeta potential of (30–150) mV (measurements not shown). The size of the unillamelar nanoliposomes was between 80 and 150 nm in diameter (Delsa Nano C, particle analyzer, Beckman Coulter, Brea, CA, USA), and the pH of the solution was 5–7.

### 2.5. Liposome Preparation and Characterization

Liposomes were prepared by Sesderma laboratories (Valencia, Spain) according to the patented procedure as previously described [26,27]. Briefly, to manufacture the sodium ascorbate liposomes, the phospholipids were dissolved in 96%ethanol, and the sodium cholate was dissolved in non-pyrogenic double-distilled water. Subsequently, both solutions were mixed. Next, a solution of sodium ascorbate at 250 mg/mL was prepared in saline, and the lipid and aqueous phases were mixed and agitated with a food mixer. A pH of 6.5 was achieved. The suspension was incubated at room temperature for 10 min and then filtered through the Minisart^®^ syringe filter with 0.2 μm pore size.

Size determination, polydispersity index, and zeta potential were measured with the Delsa Nano C Particle Analyzer (Beckman Coulter Alcobendas, Madrid, Spain).

Samples were injected into a dialysis cell (Slide-A-Lyzer^®^ Dialysis Cassettes, Pierce, NJ, USA) with a hydrophilic cellulose membrane (10,000 molecular weight cut-off [MWCO]). After 4 h of dialysis, the dialyzing medium was changed and left overnight. After 24 h of dialysis, samples were withdrawn for high-performance liquid chromatography (HPLC) analysis (the reference solution was a sample that was not dialyzed). The experiment was repeated three times for each sample.

### 2.6. Analysis of Cell Viability

The cytotoxicity of the polymers in Huh-7 and ACE2+A549 cell lines was quantified using a CellTiter 96 Aqueous Non-Radioactive Cell Proliferation Assay Kit (Promega, Madison, WI, USA) based on the MTT reagent. Non-confluent monolayers of cells plated in 96-well tissue culture plates were grown for 24 h before use. Cells were then treated for 48 h with FL or LL at concentrations ranging from 0.5 to 10^−4^% *w*/*v*. Four replicates were performed for each concentration. The cells were then incubated as indicated by the manufacturer of the kit, and the resulting colored solution was quantified using the scanning multi-well spectrophotometer iMarkTM Microplate Reader (BioRad, Hercules, CA, USA) measuring the absorbance at 595 nm. The readouts obtained from the MTT assay were further normalized to the value of untreated cells, and CC50 values were calculated.

### 2.7. Viral Assays in Cell Lines

#### 2.7.1. Antiviral Assays with HCoV-229E

The antiviral activity of free or liposomal Lf (in contact with cell cultures at all times) was assayed. Huh-7 cells were seeded in 48-well culture plates and treated for 1 h with either FL or LL at a range of concentrations that were non-cytotoxic. Then, cells were infected with HcoV-229E at an MOI of 0.5 at 33 °C in the presence of the candidate compounds. Subsequently, the virus was removed, and cells were washed with PBS and maintained in a fresh culture medium containing FL or LL in a humidified atmosphere at 33 °C. Cells were fixed for flow cytometry at 48 h p.i.

#### 2.7.2. Antiviral Assays with LV-St and LV-VSV Pseudoviruses

Regarding pseudovirus assays, ACE2+HEK293T or ACE2+A549 cells were seeded in 96-well tissue culture plates and infected with either LV-St or LV-VSV (MOI 0.1. Cells were pretreated with FL or LL for 1h, and then infected with the correspondent virus in the presence of polybrene (4 ug/ml) and the compounds. At 48 h p.i., cells were fixed for flow cytometry.

### 2.8. Viral Assays in Lung Tissue

Duplicates of five-fold serial dilutions of the four polymers were tested in human lung tissue (HLT) cells using at least three different donors. HLT cells were added at a density of 300,000 cells/well and incubated with the LL of FL for 1 h before infection. Then, an MOI of 0.1 of the VSV*ΔG(Luc)-S virus was added to the wells, and plates were spinoculated at 1200 g and 37 °C for 2 h. After the infection, fresh RPMI medium was added to the wells, and cell suspensions were transferred into a 96-well flat-bottom plate. Cells were then cultured overnight at 37 °C in a 5% CO_2_ incubator. Each plate contained the following controls: no cells (background control), cells treated with medium (mock infection), cells infected but untreated (infection control), and cells infected and treated with the drug camostat mesylate (S2874, Sigma) as a positive control [24]. After 20 h, cells were incubated with Britelite plus reagent (Britelite plus kit; PerkinElmer) and then transferred to an opaque black plate. Luminescence was immediately recorded by a luminescence plate reader (LUMIstar Omega). In parallel, drug cytotoxicity was monitored by luminescence. To evaluate cytotoxicity, the CellTiter-Glo Luminescent kit (Promega) was used. Data were normalized to the mock-infected control, after which EC50 and CC50 values were calculated.

### 2.9. Statistics

All statistical analyses were performed using GraphPad Prism (version 8.0.1, GraphPad Software, Inc., Boston, MA, USA). Data were subjected to Mann–Whitney U tests (non-parametric samples) or two-tailed Student’s t-tests (parametric samples) to determine significant differences between groups, and *p* values < 0.05 were considered statistically significant. For the CC50 and EC50 values, which indicate the concentration of the compound that leads to a 50% reduction in cell viability and viral infection, respectively, non-linear fit regression models were used (four parameters).

## 3. Results

### 3.1. Determination of Non-Cytotoxic Doses of Bovine FL and LL

**Figure 1:**
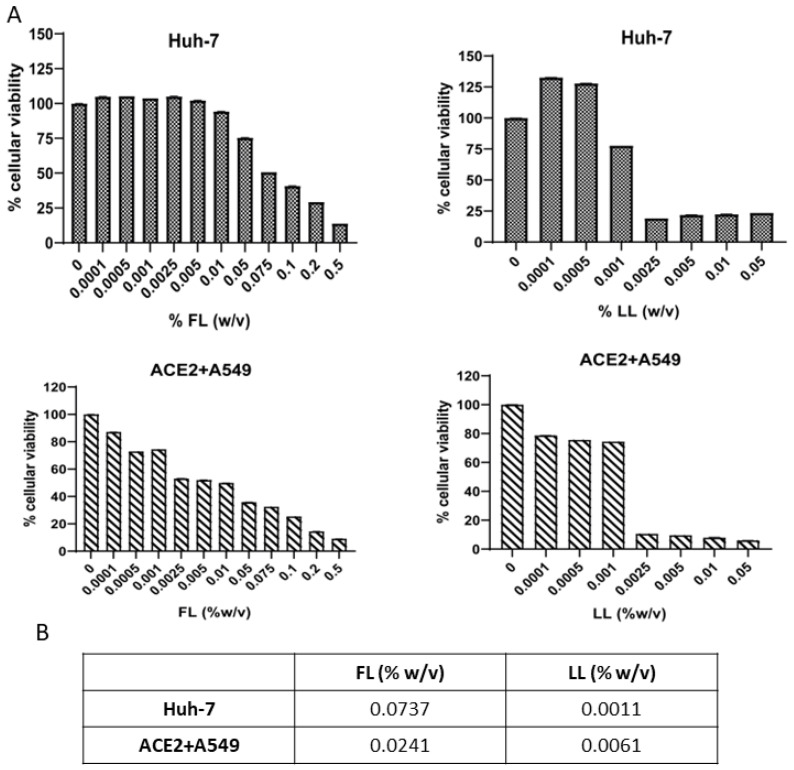
Viability of Huh-7 and ACE2+A549 cells exposed to FL and LL. Cells were cultured in culture medium and treated or mock-treated for 48 h with a range of concentrations of the compounds. (**A**) Cell viability was measured by the MTT tetrazolium salt assay and calculated as the percentage of viability compared to untreated cells; columns represent the mean viability ± S.D. (*n* = 8) after exposure to the drugs. (**B**) CC50 values for each compound and cell line. EC50 values were determined by a non-linear fit model with variable response curve (four parameters).

To determine the toxicity of FL and LL in the cell lines used in the study, an MTT assay was performed. Cells were cultured for 48 h in culture medium in the presence of different concentrations of each compound, and the viability was quantified (Figure 1A). CC50 values were calculated for each compound (Figure 1B). Both free and liposomal Lfs followed the same pattern of cytotoxicity in all lines tested. According to FL, cells maintained approximately 80% viability when treated with FL 0.01 %(*w*/*v*) in Huh-7 cells and 10^−3^%(*w*/*v*) in the ACE2+A549 cell line. Nonetheless, cells treated with LL conserved their viability at a lower concentration of 10^−3^%(*w*/*v*) in both cell lines tested. When the LL concentration was increased, the viability dropped drastically. Therefore, LL needs to be used at lower concentrations than its free form in vitro, as liposomalization increases its toxicity. To make sure that the empty liposomes alone did not exert additional cytotoxic effects or were not responsible for the antiviral effect itself, their toxicity and antiviral effect were also tested (Supplementary Figure S1). These above mentioned concentrations were the highest non-toxic concentrations of the compounds in the cell lines tested, and they were selected to use them for the following experiments.

### 3.2. Liposomal Bovine Lactoferrin Exerts Antiviral Effect In Vitro against SARS-CoV-2 Pseudoviruses and HCoV-229E

To evaluate whether bLf had an antiviral effect against SARS-CoV-2 pseudoviruses, the lung A549 cell line expressing human ACE2 was treated with FL or LL at different non-cytotoxic concentrations for 1 h prior to infection. Then, cells were infected with LV-St or LV-VSV in the presence of the compounds. Later, cells were washed and maintained in medium with either FL or LL for 48 h. The percentage of infected cells was measured by flow cytometry.

**Figure 2:**
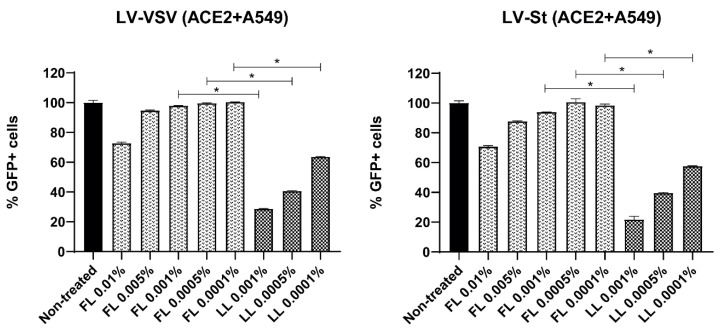
Comparison of the antiviral effect of free and liposomal bovine lactoferrin against LV-VSV and LV-St pseudoviruses in ACE2+A549 cells. The cells were incubated during all steps with either FL or LL at a specific range of concentrations and were subsequently infected with its corresponding virus at an MOI of 0.1. Columns represent the mean percentage of GFP+ cells compared to untreated cells. Triplicate experiments were performed for each data point (*n* = 3), and the value is presented as mean percentage of infection/viability ± S.D. * *p* < 0.05 was considered statistically significant.

At the same concentrations tested, LL decreased viral infection significantly more than FL (Figure 2). In addition, at very low doses (10^−4^%*w*/*v*), FL did not exert any antiviral effect, while LL managed to reduce the infection by more than 50% even at a concentration lower than 10^−3^(%*w*/*v*). Results obtained were very similar with the two different types of pseudoviruses tested: those expressing either SARS-CoV-2 S protein or VSV protein.

**Figure 3:**
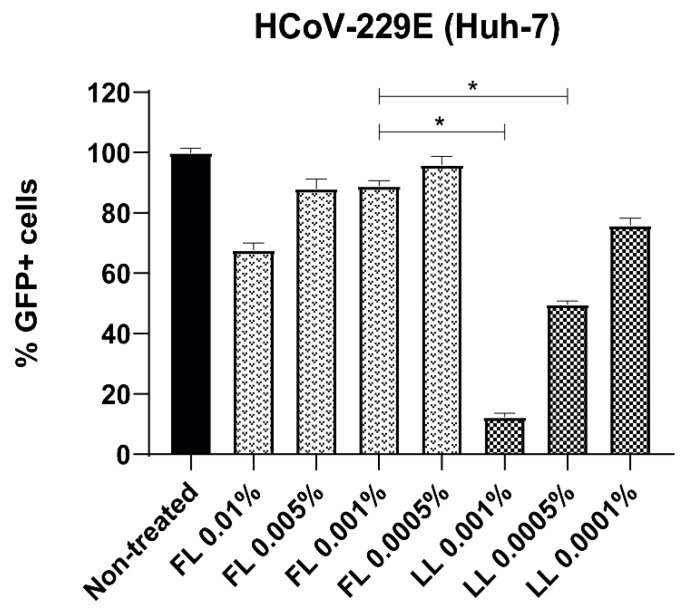
Comparison of the antiviral effect of free and liposomal bLf against HCoV229E in the Huh-7 cell line. Cells were incubated during all steps with either FL or LL at a specific range of concentrations and were subsequently infected with the corresponding virus at an MOI of 0.5. Columns represent the mean percentage of GFP+ cells compared to untreated cells. Triplicate experiments were performed for each data point (*n* = 3), and the value is presented as mean percentage of infection/viability ± S.D. * *p* < 0.05 was considered statistically significant.

The antiviral effect of bLf against HCoV-299E was also assayed. Huh-7 cells were subjected to infection in the presence of FL or LL during all steps. At 48 h p.i., cells were collected and analyzed by flow cytometry. Huh-7 cells treated with bLf showed a decrease in infection; this effect was significantly greater in those treated with LL in comparison to those treated with FL (Figure 3).

### 3.3. Antiviral Effect of Liposomal Bovine Lactoferrin in Human Lung Tissue Cells (HLT)

Following the promising results obtained in cell line models, the following step was taken to prove the antiviral activity of LL in more clinically relevant models, such as human lung tissue (HLT) cells. A rapid platform for the identification of viral entry inhibitors was used [23]. Cell suspensions from primary HLTs were extracted from three different patients with negative PCR tests for SARS-CoV-2, processed, and cultured for the assay. HLT cells were infected with VSV*∆G (Luc)-Spike virus in the presence of a 1/5 serial dilution of LL of FL. Antiviral activity and cell viability were measured by luminescence at 20 h post-exposure. As a positive control, the drug camostat mesylate was added (results not shown) due to previous reports describing high antiviral activity in this HLT model [23] and in precision-cut lung slices [28].

**Figure 4:**
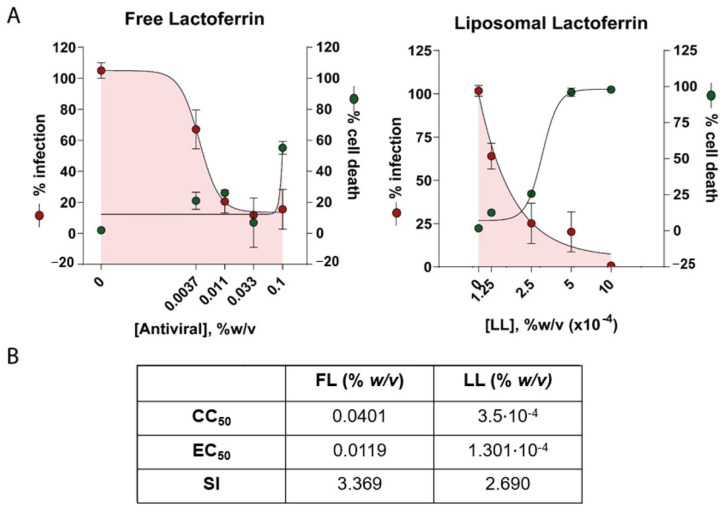
Percentage of viral entry in HLT cells exposed to VSV*ΔG(Luc)-Spike in the presence of free or liposomal bovine lactoferrin. HLT cells were incubated with VSV*∆G (Luc)-Spike virus in the presence of a 1/5 serial dilution of FL or LL. Antiviral activity and cell viability were measured 20 h p.i by luminescence. (**A**) A non-linear fit model with variable response curve (four parameters) from at least three independent experiments in replicates is shown (red lines). The cytotoxic effect on HLT exposed to drug concentrations in the absence of virus is also shown (green lines). (**B**) CC50, EC50, and selectivity index (SI) values of each compound for HLT cells. Triplicate experiments were performed for each data point (*n* = 3), and the value is presented as mean of the percentage of viral entry/viability ± S.D.

Preliminary assays revealed that the calculated EC50 and CC50 values in cell line models differed drastically from the values in HLT cells for the same concentration. Therefore, CC50 values were recalculated for this model. FL was able to reduce viral entry to approximately 80% without being cytotoxic (Figure 4A). In addition, LL managed to reduce the infection with an EC50 much lower than its free form (Figure 4B). However, HLT cells were more susceptible to LL, and its CC50 decreased in comparison to that calculated in cell models. Therefore, the effective antiviral activity of LL reported in vitro is limited in this model due to cytotoxicity issues (see selectivity index values).

## 4. Discussion

Antiviral assays demonstrated the potential activity of LL in comparison to FL against HCoV-229E and SARS-CoV-2 pseudoviruses in vitro. At non-cytotoxic doses, LL managed to reduce the infection at approximately 80% in both cell lines tested. According to the previous literature, the antiviral activity of Lf is associated with its binding to negatively charged glycosaminoglycan viral receptors (e.g., heparan sulfate proteoglycans and sialic acids), thus preventing the first contact between the virus and the cell [11,13]. Specifically, for SARS-CoV-2 infection, Lf was shown to bind directly to viral envelope proteins, such as the S protein [29]. Therefore, Lf mainly exerts its effect in the early phase of infection.

Regarding the liposomal form of bLf, LL demonstrated a greater antiviral effect in comparison to its free form in vitro against HCoV229E and SARS-CoV-2 pseudoviruses. Liposomalization of Lf provides a delayed release of the compound in the target organ, the respiratory tract. Although Lf is hydrophilic, when encapsuled, it behaves as amphiphilic, a characteristic that allows this form to interact with the natural surfactants of the target tissue [9,17]. Encapsulation also prevents LL from being rapidly digested by the enzymes and acids of the stomach after oral administration. In addition, as Lf is reported to attach to the ACE2 cell receptor used by SARS-CoV-2 to enter the cell, the components of the liposome may induce protein changes in cellular TMPRSS2, which is also involved in the entry of this virus [15]. The mechanism of action of Lf against SARS-CoV-2 is also explained by its direct interaction with the receptor binding domain (RBD) of the Spike protein [30,31] and its binding to heparan sulfate proteoglycans, thus preventing the early attachment phase [14]. In addition, some in silico studies showed that Lf also interacts with the transferrin receptor 1, which can be an alternative co-receptor for SARS-CoV-2 entry [31,32].

A recent study showed that LL was proven to be more effective in diminishing SARS-CoV-2 S protein binding to HaCaT cells than FL in vitro [15]. Considering the results obtained, LL could perhaps be considered for intravenous administration or nebulization [33]. The latter route is ideal for lactoferrin to trap airborne coronaviruses at the respiratory tract level [34] by accessing pulmonary alveoli. As viral replication could continue for several weeks in the case of critically ill patients, repetitive administration should be carried out. Its low cost, affordability, and easy production in comparison to other drugs tested against SARS-CoV-2, coupled with the fact that it is already approved, makes [35] LL an attractive therapeutic option to be quickly tested in clinical trials. It may have therapeutic potential not only for this virus but also for other airborne viruses that attack the naso-oropharyngeal pathways. Lf is rapidly extracted from bovine milk and is used in several commercial products such as infant formula, nutritional supplements, and body lotions [10].

In addition to its antiviral properties, lactoferrin can modulate the immune response mainly by two mechanisms: enhancing the antigen expression of B cells and regulating T cells [36]. Considering that mortality from COVID-19 is not simply due to viral infection but is in some cases associated with a cytokine storm that leads to acute respiratory distress, lactoferrin might promote the expression of type I interferons and anti-inflammatory cytokines (TGF-β or IL-10), thus counteracting the activation of this cytokine storm [37,38].

Nevertheless, in the HLT model used for characterization of viral entry inhibitors, LL prevented the viral entry of VSV*∆G (Luc)-Spike virus but with a low SI (2.69). The window between cytotoxicity and antiviral activity is not high enough [39] to consider LL an exceptional drug for treatment. Nonetheless, a recent study reported that an LL nutritional syrup administered to SARS-CoV-2-infected patients resolved symptoms of COVID-19 [9]. It must be taken into account that HLT, despite its speed and similarities to the real lung environment, lacks reproducibility, as each experiment starts with lung samples from different donors [23].

These findings on the in vitro antiviral activity of LL against the viruses tested in this study provide additional evidence of the use of LL and the advantages of liposomalization. Further research is needed to completely unravel the mechanism of action of LL, and studies on the route of delivery and its administration alone or combined with other antiviral drugs in patients suffering from respiratory viruses are needed.

## Data Availability

The data presented in this study are available on request from the corresponding author, upon reasonable request.

## Supplementary Materials

The following are available online at https://www.mdpi.com/article/10.3390/v15040972/s1, Figure S1: Cell toxicity and antiviral capacity of empty liposomes solution.
