# Supplementary material for: Liposomal Lactoferrin Exerts Antiviral Activity against HCoV-229E and SARS-CoV-2 Pseudoviruses In Vitro
Source: Viruses. 2023 Apr 15;15(4):972. doi: 10.3390/v15040972 (PMC10142420; doi:10.3390/v15040972)
Supplement: Supplementary file 1 [file viruses-15-00972-s001.zip › viruses-2324940-supplementary.pdf]

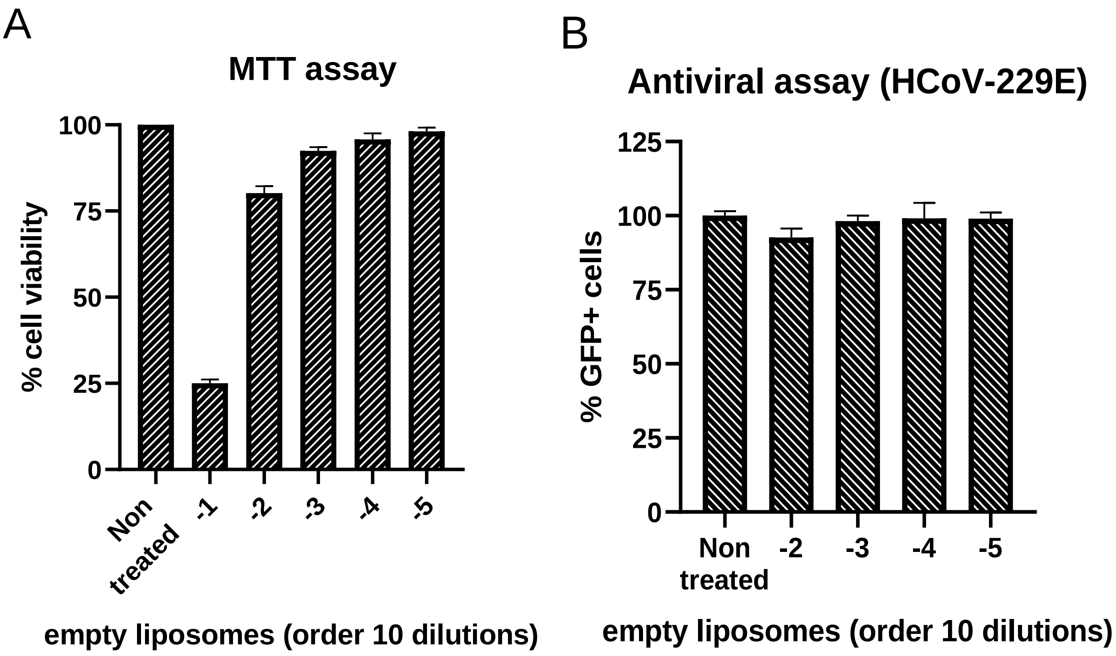


**Figure S1. Cell toxicity and antiviral capacity of empty liposomes solution.** A) Viability of Huh-7 cells exposed empty liposomes. Cells were cultured in culture medium and treated or mock-treated for 48 h with order 10 dilutions of the liposomal structure. Cell viability was measured by MTT tetrazolium salt assay and calculated as the percentage of viability compared to untreated cells; columns represent the mean viability ± S.D. ($n$ = 3) after exposure to the drugs. B) Antiviral assay of empty liposomes against HCoV229E in Huh-7 cell line. The cells were incubated during all steps with order 10 dilutions of empty liposomes and subsequently infected with its corresponding virus at a MOI of 0.5. Columns represent the mean percentage of GFP+ cells compared to untreated cells
